# First Reported Complete Genome Sequence of a Dengue Virus Serotype 4 Strain from Papua New Guinea

**DOI:** 10.1128/MRA.01082-18

**Published:** 2018-09-27

**Authors:** Alyssa T. Pyke, Peter R. Moore, Glen Hewitson, Peter Burtonclay

**Affiliations:** a Public Health Virology Laboratory, Forensic and Scientific Services, Coopers Plains, Queensland, Australia; University of Maryland School of Medicine

## Abstract

A male patient in his 50s who traveled from Papua New Guinea (PNG) to Australia in 2016 was diagnosed with a dengue virus serotype 4 (DENV-4) infection, and the virus was isolated from his acute-phase serum. Here, we describe the first complete genome sequence of a DENV-4 strain from PNG.

## ANNOUNCEMENT

In view of the ubiquity of the mosquito-borne dengue viruses (DENVs) within tropical and subtropical regions and the frequency of large-scale DENV epidemics, almost 50% of the world’s population (3.9 billion people) is considered at risk for DENV infection (1, 2), and approximately 25% (96 million) of the estimated 390 million infections per year are clinically apparent (3). Dengue disease can range from a mild febrile illness to a life-threatening condition, including dengue hemorrhagic fever (DHF) or dengue shock syndrome (DSS) (4).

There are four genetically and antigenically distinct DENV serotypes (DENV-1 to DENV-4) (genus Flavivirus, family Flaviviridae), and transmission in urban cycles involves primarily Aedes aegypti or Aedes albopictus mosquitoes (5–7). DENV genomes are 5′-capped, positive-sense, single-stranded RNA molecules (~11 kb) which encode a single, large open reading frame (ORF) flanked by 5′ and 3′ terminal untranslated regions (UTRs). The ORF is translated into three structural and seven nonstructural proteins (8, 9).

Dengue is endemic in the Western Pacific where reported case numbers have escalated from ~200,000 in 2008 to ~450,000 in 2015 (10). Located in the southwestern Pacific, Papua New Guinea (PNG) has been a major source of DENVs causing outbreaks in north Queensland, Australia (11–13). Recently, we demonstrated cocirculation of DENV-1 to DENV-4 strains in PNG and the evolution of new genetic lineages (12).

In mid-2016, a male in his 50s traveled from PNG to north Queensland, presenting with fever, chills, rigors, headaches, myalgia, rash, and diarrhea. DENV-4 RNA was detected (threshold cycle number, 23) in a serum sample (collected on day 0 of symptom onset) by reverse transcription real-time polymerase chain reaction (RT-rtPCR) (14, 15), and a DENV-4 isolate (strain PNG 2016a) was recovered from the sample. Ethical approval for this study was granted by the Forensic and Scientific Services Human Ethics Committee.

Based on the envelope gene nucleotide sequence (GenBank accession number KY427082), we previously reported the phylogenetic grouping of strain PNG 2016a within DENV-4 genotype II (12). Here, we describe the whole-genome sequencing (WGS) of PNG 2016a, the first reported complete genome sequence of a PNG DENV-4 strain. Briefly, strain PNG 2016a RNA was extracted from C6/36 cell culture supernatant, passage 1, and circularized using a tobacco decapping enzyme (Enzymax LLC, USA) and a T4 RNA ligase (New England Biolabs, USA) as reported previously (16). Next, we performed WGS (Illumina). The cDNA libraries were constructed using the Nextera XT kit and then sequenced using the V2 mid-output kit on a NextSeq 500 machine as previously described (17). More than 6 × 10^6^ reads were obtained for each sample (paired at 2 × 151 nucleotides [nt]) and assembled by mapping reads to a reference genome (GenBank accession number KJ596658) using Geneious R10 (version 10.2.3) software and default parameters with the low sensitivity setting. A BLASTN analysis and comparison of the complete genome of strain PNG 2016a (10,653 nt; G+C content of 47.0%) with available DENV GenBank sequences demonstrated 99.3% nt sequence identity with an Indonesian 2015 DENV-4 strain (GenBank accession number KU523872). Only a limited number of PNG DENV sequences have been described, and this is the first reported complete genome sequence of a PNG DENV-4 strain. These data will advance current knowledge of DENV diversity and molecular evolution, particularly in the Australasian region.

### Data availability.

Raw sequencing reads were deposited into the Sequence Read Archive under accession number SRP159163. The consensus genome sequence was deposited in GenBank under accession number MH382789.
